# Role of ROS-Induced NLRP3 Inflammasome Activation in the Formation of Calcium Oxalate Nephrolithiasis

**DOI:** 10.3389/fimmu.2022.818625

**Published:** 2022-01-27

**Authors:** Yunlong Liu, Yan Sun, Juening Kang, Ziqi He, Quan Liu, Jihua Wu, Derong Li, Xiang Wang, Zhiwei Tao, Xiaofeng Guan, Wusheng She, Hua Xu, Yaoliang Deng

**Affiliations:** Department of Urology, First Affiliated Hospital of Guangxi Medical University, Nanning, China

**Keywords:** reactive oxygen species, inflammasome, macrophage, nanoparticles, endoplasmic reticulum stress, autophagy

## Abstract

Calcium oxalate nephrolithiasis is a common and highly recurrent disease in urology; however, its precise pathogenesis is still unknown. Recent research has shown that renal inflammatory injury as a result of the cell-crystal reaction plays a crucial role in the development of calcium oxalate kidney stones. An increasing amount of research have confirmed that inflammation mediated by the cell-crystal reaction can lead to inflammatory injury of renal cells, promote the intracellular expression of NADPH oxidase, induce extensive production of reactive oxygen species, activate NLRP3 inflammasome, discharge a great number of inflammatory factors, trigger inflammatory cascading reactions, promote the aggregation, nucleation and growth process of calcium salt crystals, and ultimately lead to the development of intrarenal crystals and even stones. The renal tubular epithelial cells (RTECs)-crystal reaction, macrophage-crystal reaction, calcifying nanoparticles, endoplasmic reticulum stress, autophagy activation, and other regulatory factors and mechanisms are involved in this process.

## Introduction

One of the most frequently encountered diseases in urology is kidney stones, which are closely related to multiple factors such as environment, genetics, and metabolic abnormalities; however, its exact pathogenesis is still unclear (1). Over the past few years, the incidence of kidney stones has been increasing year by year, with the incidence rate reaching 8.8% in the United States and 3.5−7.4% in Asia (2, 3). In addition, the recurrence rate of kidney stones is as high as 50% within 5−10 years following drug therapy or surgical treatment, and the recurrence rate within 20 years is even higher at 75% (4). Because of the high incidence and recurrence rate, kidney stone disease does not only greatly affects human life and health, but also significantly increases the financial burden of the healthcare system (5). Therefore, exploring the exact pathogenesis of kidney stones and finding new therapeutic targets has important theoretical significance and application value in the treatment and prevention of stones.

It is known that kidney stone formation involves a series of complicated processes, including urine supersaturation, calcium salt crystal adhesion, accumulation, nucleation, growth, and retention in the kidney (6). Common crystals in the kidney mainly include oxalate (Ox), calcium oxalate (CaOx), calcium phosphate, and urate, among which CaOx crystals are the most common (7). The retention and subsequent growth of crystals within the kidney are inseparable from kidney stone formation. A large amount of research has shown that the cell-crystal-induced inflammatory response plays a crucial role in the formation of CaOx kidney stones (8–10). In this process, the massive generation of reactive oxygen species (ROS) and the activation of NLRP3 inflammasomes promote the maturation and discharge of inflammatory factors, leading to inflammation in the kidneys and renal tubular epithelial cells (RTECs) (11, 12). In this review, we will describe in detail the role of inflammation arising from ROS-induced NLRP3 inflammasome stimulation in the formation of CaOx kidney stones.

## ROS-Induced Activation of NLRP3 in RTECs-Crystal Reaction

ROS refers to a series of active oxygen clusters and their metabolites produced by organisms in the process of aerobic metabolism, and includes superoxide anion (O^2-^), hydrogen peroxide (H_2_O_2_), hydroxyl radical (OH-), etc., which are mainly produced on the electron respiration chain of mitochondria under the catalysis of nicotinamide adenine dinucleotide phosphate (NADPH) oxidase (13, 14). Due to its high biological activity, ROS have a crucial role in the pathophysiological process of many diseases (15–17). Many studies have shown that the origination of CaOx kidney stones is intricately associated with the excessive production of ROS mediated by oxidative stress in RTECs (18–20). Clinical studies have confirmed that the levels of malondialdehyde (MDA), thiobarbituric acid reactive substances (TBARS), α-glutathione-s-transferase (α-GST), β-galactosidase (GAL), and N-acetyl-β-D-glucosaminidase (NAG) in the urine of CaOx nephrolithiasis patients are significantly higher than those of healthy patients, suggesting that the development of renal calculi is related to RTEC injury induced by ROS (20). In our previous study, we found that when taurine, N-acetylcysteine (NAC), catalase, or superoxide dismutase (SOD) were added to the rat CaOx kidney stone model, the activity of NADPH oxidase and the production of ROS and LDH decreased, the renal tissue and cell damage and inflammatory reaction triggered by Ox and/or CaOx crystals were alleviated, the deposition of crystal in the kidney was significantly decreased, and the formation rate of CaOx kidney stones was reduced (21). In further experimental studies, we found that oxalic acid or CaOx crystals could prompt oxidative stress in RTECs. The production of a large number of ROS initiates the NF-κB signal pathway, promotes the secretion of a series of cytokines, and induces inflammation, resulting in RTEC degeneration, injury, exfoliation, and basement membrane exposure, which creates conditions favorable for the conservation of crystals in the lumen of renal tubules, and is ultimately conducive to the development of CaOx kidney stones (22, 23).

Inflammasome is a multiprotein complex that is triggered and activated after cells recognize dangerous-associated molecular patterns (DAMPs) for instance, monosodium urate and silica, and pathogen-associated molecular patterns (PAMPs) such as viruses and bacteria (24). The main members of the inflammasome family are NOD-like receptors (NLRs), AIM2-like receptors, and pyrin inflammasome. Of these, NLRP3 inflammasome is the most studied multiprotein complex, and mainly consists of NLRP3, caspase-1, and apoptosis-associated spot-like protein (ASC) (25, 26). After endogenous or exogenous stimulation, activated NLRP3 can recruit ASC protein and activate caspase-1, subsequently induce the maturation and release of inflammatory factors such as interleukin (IL)-1β and IL-18, and participate in a variety of inflammatory responses in the body, which has become a hot topic in the study of inflammatory mechanisms (27). Previous research has indicated that CaOx crystals can directly stimulate the secretion of IL-1β by renal dendritic cells *via* the NLRP3/ASC/caspase-1 axis, in addition to damaging RTECs to release ATP, indirectly activating the NLRP3 inflammasome, and then stimulating the secretion of IL-1β by dendritic cells, resulting in renal inflammatory response and inflammatory injury of RTECs (28). Studies have also shown that after knockout of NLRP3, the rate of renal stone formation in mice, who were fed a high oxalic acid diet, as well as caspase-1 expression and IL-1β secretion in RTECs all decreased significantly (8, 10, 29). Furthermore, the induction of NLRP3 inflammasome can participate in the formation of renal stones by changing the adhesion of cells to crystals. Khan and other researchers have found that ROS can up-regulate the expression of hyaluronic acid (HA), osteopontin (OPN), and CD44 *via* the p38MAPK pathway, and change the adhesion of RTECs to CaOx crystals as well as stimulate the formation of CaOx stones (30). Qi et al. (31) found that the induction of the NLRP3 inflammasome plays a connective role in the process of ROS changing the cell adhesion to crystals and promoting stone formation through the p38MAPK signaling pathway, and related proteins such as phosphorylated p38 and c-Jun play an important regulatory role. Research by Joshi et al. (10) has indicated that in NLRP3 gene-deficient mice, who received a high oxalate diet, the expression levels of OPN, HA, and CD44 on the surface of RTECs were significantly decreased, and the formation speed of CaOx stones was significantly lower compared to that of normal mice.

It has been established that ROS are a crucial component in the activation of NLRP3 inflammasomes (32, 33). The application of ROS inhibitors can impede the activation of NLRP3 inflammasomes, alleviate cell inflammatory damage, and delay progression of the disease (34, 35). *In vivo* as well as *in vitro* studies have shown that CaOx crystals are capable of inducing the generation of ROS in the kidney, and then activating NLRP3 inflammasome to cause inflammatory damage to RTECs and renal tissue, thus promoting the formation of CaOx stones (10). We found in our previous research that atorvastatin treatment downregulated ROS production, suppressed the induction of NLRP3 inflammasome pathways, decreased the release of IL-1β, IL-6, IL-18, and tumor necrosis factor-α (TNF-α), and ameliorated inflammatory injury and crystal deposition induced by CaOx crystals in kidney tissues of rats and HK-2 cells (36). Therefore, we believe that oxalic acid or CaOx crystals induce ROS production in RTECs during the formation of CaOx renal calculi and mediate the activation of NLRP3 inflammatory corpuscles. This process leads to inflammatory cell infiltration and RTEC degeneration and necrosis, and causes a renal inflammatory cascade effect, promotes the adhesion, accumulation, nucleation, and secondary growth of CaOx crystals, ultimately resulting in the formation of renal stones.

## ROS-Induced Activation of NLRP3 in Macrophage-Crystal Reaction

In the cell-crystal inflammatory response, macrophages, as an important inflammatory cell with phagocytic function, participate in CaOx crystals formation in the kidney (37). The expression of macrophage-related genes in renal papillary calcified plaque tissues was found to be significantly higher in patients with kidney stones than in patients without (38). The results that have been reported in *in vitro* studies show that under the stimulation of CaOx crystals, kidney cells produce a variety of inflammatory factors, which induce monocytes or macrophages to migrate to the place where the stone crystals are deposited and transported through pinocytosis. At the same time, the crystals can promote the expression of NADPH oxidase in the cell to generate a large amount of ROS, induce the nuclear factor κB signal transduction pathway, and produce a great amount of inflammatory factors including TNF-α, IL -6, and IL-1β. This further causes inflammatory damage to kidney cells, leading to cell degeneration, necrosis, exposure of basement membrane, etc., which is conducive to the formation of renal calcification plaques (39, 40). In the hyperoxaluria rat model, hyperoxaluria can activate the NF-κB signal transduction pathway through ROS, induce the kidney to express a great amount of inflammatory factors including MCP-1, IL-6, and IL-1β. Following these processes, inflammatory cell infiltration (such as macrophages) and renal interstitial damage occur, creating conditions for CaOx crystal formation in the kidneys (41). This indicates that the ROS-mediated macrophage-crystal inflammatory response plays a key role in the formation and development of CaOx kidney stones.

In the resting state, the expression level of NLRP3 in dendritic cells, macrophages, and other innate immune cells is relatively low, but after endogenous and exogenous stimulation, activated NLRP3 can recruit the ASC protein, activate caspase-1, and then induce the maturation and release of inflammatory factors including IL-18 and IL-1β (27). It has been reported that macrophages can cause intracellular lysosomes to rupture after they internalize pathogens or crystals. After the lysosome ruptures, the protease (such as cathepsin B) in the lysosome is released into the cytoplasm (42), leading to the opening of effector cell membrane ion channels, causing changes in intracellular ion concentration, which promotes the activation of NLRP3 inflammasomes. Hydroxyapatite crystals can activate the NLRP3 receptor through ROS to stimulate human monocyte macrophages and rat macrophages to produce the inflammatory factor IL-1β (43). Macrophages activate the NADPH enzyme to produce ROS after engulfing CaOx crystals and accumulate in the kidneys, leading to aggravation of OS; after which ROS can activate inflammatory pathways such as NLRP3 and TLR4 (44). Blocking NLRP3 may protect macrophages from oxalate damage (45). Therefore, we speculate that after macrophages engulf CaOx crystals, up-regulation of ROS may mediate the activation of NLRP3 inflammasomes, induce the secretion of inflammatory factors, and cause aggravation of the inflammatory damage in kidney tissues and RTECs, thereby promoting the formation process of CaOx kidney stones.

High mobility group box 1 (HMGB1) is an important inflammatory mediator and inflammatory cytokine and is widely present in various cells. It can be actively or passively released by activated mononuclear macrophages and damaged or necrotic cells to trigger the biological effect of initiating, maintaining, and enhancing the inflammatory response (46). Studies have found that the application of anti-HMGB1 antibodies can significantly reduce the infiltration of renal tubular interstitial neutrophils and monocytes, alleviate the inflammatory damage of RTECs, and inhibit the release of inflammatory factors in kidney tissues such as MCP-1, TNF-α, and IL-6. By blocking the inflammatory cascade mediated by HMGB1, the renal function can be improved and renal ischemia-reperfusion injury reduced (47). Wang et al. (48) reported that in patients with calcium-containing kidney stones, an increased expression of HMGB1 and MCP-1 was found in their urine. The results of *in vitro* studies show that high calcium ions stimulate RTECs to activate the HMGB1-RAGE/TLR4-NF-κB signaling pathway and stimulate the secretion of inflammatory factors TNF-α, IL-1β, and IL-6 (49). Moreover, previous studies have also indicated that there is a close relationship between the secretion of HMGB1 and the activation of NLRP3 inflammasomes (50, 51). Lamkanfi et al. (46) found that lipopolysaccharide-induced secretion of HMGB1 by activated macrophages requires the participation of the NLRP3, ASC, and caspase-1 inflammatory complex.

In summary, we speculate that after macrophages engulf CaOx crystals, ROS generated by oxidative stress in the cells stimulate macrophages by activating NLRP3 inflammasomes, inducing increased secretion of HMGB1, and activating the NF-κB signal transduction pathways to stimulate macrophages to secrete inflammatory factors including TNF-α and IL-1β. On the other hand, HMGB1 itself can in turn activate the NLRP3 inflammasome, forming a positive feedback effect of inflammation, and thereby maintaining or even amplifying the inflammatory cascade, which causes severe inflammatory damage to the kidney. This promotes the adhesion, accumulation, nucleation, and subsequent growth of CaOx crystals, and ultimately leads to crystals and even stones being formed in the kidney.

## ROS-Induced Activation of NLRP3 in Calcifying Nanoparticles

Randall plaque is a kind of calcified tissue located under the mucosa of the renal papilla (9). It originates from the basement membrane of the tubular epithelial cells in the slender part of the medullary loop, gradually extends to the interstitium of the renal medulla, and finally deposits in the renal papilla’s interstitial tissue (52). Matlaga et al. (53) used endoscopic techniques to observe renal papillary plaques in 23 patients who had idiopathic CaOx stones and found that there were Randall plaques in 24 kidneys (24/46) and 156 renal papillae (156/172) indicating that most patients with CaOx stones have Randall plaques in their kidneys. Near the tubule basement membrane, the main constituent of Randall’s spots is calcium phosphate. As it gradually migrates to the lumen of renal tubules, the components of the plaque shows an obvious migration and transformation phenomenon in the form of “calcium phosphate→calcium phosphate and CaOx→CaOx” (1). Scholars believe that the secretion of hydrogen in the distal renal tubules can acidify the urine, which causes the ion activity of calcium phosphate to decrease significantly, and promotes the dissolution of calcium phosphate in the tubules to release calcium and phosphorus ions (54). In the final urine at the end of the collecting tubule, calcium ions integrate with oxalate ions to form CaOx (55).

With the development of modern molecular biology and cell biology technology, Kumar et al. (56) discovered that there are many round or quasi-circular electronic high-density small bodies that are very similar to calcifying nanoparticles (CNPs) in the Randall plaque. Although it remains to be confirmed whether these high-density bodies are CNPs, some scholars have shown that the characteristics of the thin apatite shell on the surface of CNPs are highly consistent with the Randall patch structure at the initial stage (57). CNPs can be cultured in the tissues around Randall’s spots, stone specimens, and renal pelvic urine of patients with kidney stones (58). García et al. (59) found that the rat kidney stone model can be successfully constructed by intravenous injection or direct intra-renal injection of CNPs. Furthermore, Ciftcioglu et al. (60) verified the expression of CNPs in renal calcification plaques in human kidney tissue specimens through a variety of methods, and believed that CNPs are one of the main causes of renal calcification plaques. Therefore, we can infer that there is a close correlation between Randall’s spot and CNPs.

CNPs-mediated inflammation is closely related to kidney stone formation. Our previous research found that in the co-culture system of CNPs and RTECs, RTECs can be observed to adhere to and phagocytize CNPs, resulting in an increased NADPH oxidase activity, stimulating cells to produce a large amount of ROS, leading to RTEC inflammatory damage, which in turn can cause cell death in severe cases (61). Wu et al. (58) reported that CNPs can cause mitochondrial damage after entering the cell, and then generate ROS, which mediates the damage of RTECs and the formation of stones through the ROS-JNk signaling pathway. After Shiekh et al. intravenously injected CNPs into Wistar rats, calcium deposits were observed in the pathological sections of kidneys, while inflammatory cell infiltration and aggregation were seen in the renal medulla and cortex of rats (62). After the aggregated CNPs are swallowed by macrophages, they can induce mitochondrial damage and generate ROS, which leads to the activation of caspase-1 to mediate the secretion of IL-1β, resulting in an inflammatory response (63). As the effector protein of NLRP3 inflammasome, caspase-1 is responsible for cutting the inactive pro-inflammatory cytokine pro-IL-1β into mature IL-1β (64). We speculate that CNPs may activate NLRP3 inflammasomes through the production of ROS, thereby mediating the cell-crystal inflammatory response, leading to renal tissue inflammatory damage, inducing calcium phosphate-CaOx heterogeneous nucleation, and ultimately leading to the formation of intra-renal crystals.

## ROS-Induced Activation of NLRP3 in Endoplasmic Reticulum Stress

The endoplasmic reticulum (ER) is an important organelle widely present in mammalian cells. It is the main intracellular site for protein manufacture and processing, and the main calcium storehouse for maintaining calcium homeostasis (65). When the body is stimulated by glucose deficiency, oxidative stress, and Ca^2+^ metabolic disorders, the imbalance of ER protein dynamics leads to ER stress, and as a result, initiates the unfolded protein response (UPR), and participates in the pathophysiological process of many diseases (66). During observations under the electron microscope, our team accidentally discovered that a sequence of abnormal morphological changes in the ER, for instance, enlargement and malformation, appeared in the kidney cells of CaOx kidney stone rats (67). Further research has shown that ER stress is related to the development of CaOx kidney stones (67, 68), which is consistent with the results as reported by Yang (69) and other research teams. Much evidence shows that the regulation of CaOx kidney stone formation by ER stress is inextricably connected to the accumulation of intracellular ROS and the activation of NLRP3 inflammasome (10, 70).

ROS are viewed as a sign of oxidative stress. Studies have reported that ROS have twofold functions in signaling ER stress. During ER stress, NADPH oxidase (NOX), present in the ER, can stimulate the production of ROS, which in turn can regulate the UPR and reinstate ER homeostasis (71). Nevertheless, if the strong stimulus continues or is not removed timely, the ER pressure cannot be relieved, and ER oxidase 1 (ERO1) will partially trigger an elevation in ROS (72). Excessive ROS production in the ER leads to the deposition of calcium in mitochondria and increases the damage in mitochondria (73). We found in previous studies that SOD can alleviate the stress of ROS, and inhibiting SOD will increase the accumulation of ROS, which in turn aggravates ER stress and promotes the formation of kidney stones (74). It is currently known that the stimulation of ROS in the presence of oxidative and ER stress is crucial for the induction of NLRP3 inflammasomes in macrophages. Aside from NOX4 activating NF-κB *via* ROS, which mediates the activation of NLRP3, it can also activate mitogen-activated protein kinase (MAPK) to stimulate the release of pro-inflammatory factors (75). Furthermore, NOX2 can regulate the expression of dsRNA-activated protein kinase R (PKR) during ER stress (76). The autophosphorylation of PKR results in the *de novo* binding of NLRP3, caspase-1, and ASC, which increases the activation of inflammasomes. The lack of protein kinase receptors significantly suppresses the release of HMGB1, IL-1β, and IL-18 (50).

In addition, Ca^2+^ released by the ER may be a general stimulus that results in activation of the NLRP3 inflammasome (77). The ER is the primary organelle where Ca^2+^ is stored, and Ca^2+^ mobilization plays a key role in the activation of NLRP3 inflammasomes. Excessive Ca^2+^ release results in an overload of mitochondrial calcium and mitochondrial damage. The accumulation of mitochondrial ROS increases, which leads to additional activation of inflammasomes and IL-1β production. Blocking Ca^2+^ mobilization can suppress the generation and activation of the NLRP3 inflammasome complex (78, 79). The C/EPB homologous protein (CHOP), a transcription factor considered to be a regulator of ER Ca^2+^ release during ER stress, can regulate the release of ER Ca^2+^ through the inositol 1,4,5-trisphosphate receptor. Under ER stress, loss of CHOP leads to a weakened Ca^2+^ release from the ER, which reduces ROS and improves cell survival (80–82). Therefore, CHOP and ER stress are thought to be a potential mechanism for amplifying the activity of NLRP3 inflammasomes to increase the inflammatory response (83).

In summary, we believe that ER stress may be an upstream or intermediate mediation mechanism of the ROS-NLRP3 signaling pathway that induces CaOx nephrolithiasis. This mechanism is not directly impacted by the IRE1, PERK, and ATF6 pathways in the classic UPR pathway (84). However, it may directly impact the expression of the terminal signal in the UPR and play a role by generating ROS or mediating Ca^2+^ mobilization.

## ROS-Induced Activation of NLRP3 in Autophagy

Autophagy is a very well conserved intracellular degradation pathway. In the process of starvation, hypoxia, or oxidative stress, cells use lysosomes to degrade damaged macromolecular proteins or organelles, which can maintain intracellular environmental homeostasis and adapt to microenvironmental changes (85, 86). Studies have shown that an intimate association exists between autophagy and inflammatory responses (87). Autophagy can mitigate the inflammatory response by clearing inflammatory protein aggregates and down-regulating the release of pro-inflammatory cytokines. Conversely, excessive activation of autophagy can stimulate the inflammasomes to release a great number of inflammatory factors and accelerate the progression in inflammatory response. It has been reported that in a renal ischemia-reperfusion injury rat model, autophagy activation can downregulate the expression of pro-inflammatory factors HMGB1, TNF-α, and IL-6, increase the release of anti-inflammatory factor IL-10, and reduce renal inflammatory injury (88). Kirkland et al. (89) revealed that excessive accumulation of intracellular ROS can directly cause a certain degree of inflammatory damage to cells. In addition, a high concentration of ROS can induce excessive activation of autophagy and directly cause cell death (90).

ROS and inflammatory responses can not only activate autophagy, but also play an important regulatory role in the formation of CaOx kidney stones. The results of our previous studies have indicated that a significantly higher level of renal autophagy can be observed in patients with CaOx kidney stones than those with normal kidneys. CaOx crystals can induce the production of ROS in tubular epithelial cells, which mediates autophagy overactivation, whereas suppression of autophagy can effectively ameliorate CaOx crystal-induced tubular epithelial cell damage and decrease renal injury and CaOx crystal deposition caused by ethylene glycol, thereby reducing the rate of kidney stone formation (90, 91). Duan et al. (92) also found that chloroquine, an autophagy inhibitor, may reduce oxidative stress damage, mitochondrial damage, and lower excretion of urinary oxalate and kidney crystal deposition in rats by suppressing the activation of the p38 signaling pathways and the expression of the renal oxalate transporter SLC26A6, ultimately suppressing the formation of CaOx crystals in rat kidneys. Sun et al. (93) performed *in vitro* and *in vivo* experiments and revealed that application of the antioxidant taurine can alleviate the oxidative stress injury of RTECs caused by CaOx crystals. The responsible mechanism is inhibition of the excessive activation of autophagy mediated by ROS through up-regulation of the Akt/mTOR signal transduction pathway by taurine. Therefore, ROS mediated autophagy also plays an important role in the formation of CaOx kidney stones.

Autophagy plays a bidirectional regulatory role in diseases associated with activation of the NLRP3 inflammasome. Saitoh (94) et al. reported for the first time in 2008 that autophagy can regulate inflammasome activation, and that LPS could induce activation of inflammasome in macrophages after knockout of the autophagy regulation gene Atg16L1, suggesting that inhibition of autophagy can stimulate the maturation and release of inflammatory factors IL-18 and IL-1β. Ko et al. (95) reported that applying the autophagy activator rapamycin to increase the level of autophagy can result in inhibition of NLRP3 inflammasome activation and its mediated inflammatory response by eliminating ROS in mitochondria. However, other studies have revealed that the excessive activation of ROS mediated autophagy can promote NLRP3 inflammasome activation and IL-1β production. Zhang (96) has shown that mechanical ventilation can mediate excessive activation of autophagy after stimulating the generation of mitochondrial ROS in pulmonary macrophages, and the inflammatory injury in the lung as a result of mechanical ventilation is caused by the activation of NLRP3 inflammasomes, which is mediated by autophagy signals and the secretion of pro-inflammatory cytokines such as IL-18 and IL-1β in pulmonary macrophages. A study by Qiu et al. (97) has indicated that arsenic trioxide (As_2_O_3_) can induce excessive autophagy in hepatocytes and stimulate the activation of NLRP3 inflammasome, while the antioxidant taurine can ameliorate the inflammatory response of hepatocytes induced by As_2_O_3_ by inhibiting the autophagy-inflammasome pathway. Moreover, inducing the NLRP3 inflammasome can also inhibit or promote autophagy. By activating inflammasomes, especially NLRP3 inflammasomes, the level of mitochondrial autophagy can be inhibited and the self-clearance of mitochondria affected, thus promoting the occurrence and development of diseases (98). Allaeys (99) et al. reported that sodium urate crystals can positively manage the formation of autophagosomes in cells by up-regulating NLRP3 inflammasome activation.

Therefore, we speculate that the excessive activation of autophagy mediated by mitochondria-derived ROS could trigger the NLRP3 inflammasome pathway, up-regulate the secretion of inflammatory factors IL-18 and IL-1β, which leads to inflammatory cell invasion and renal interstitial inflammatory injury in the course of CaOx kidney stone formation. The activation of NLRP3 inflammasome may further increase the level of autophagy, resulting in an inflammatory chain reaction in the kidney, and accelerating the formation process of nephrolithiasis.

## Summary and Perspectives

**Figure 1:**
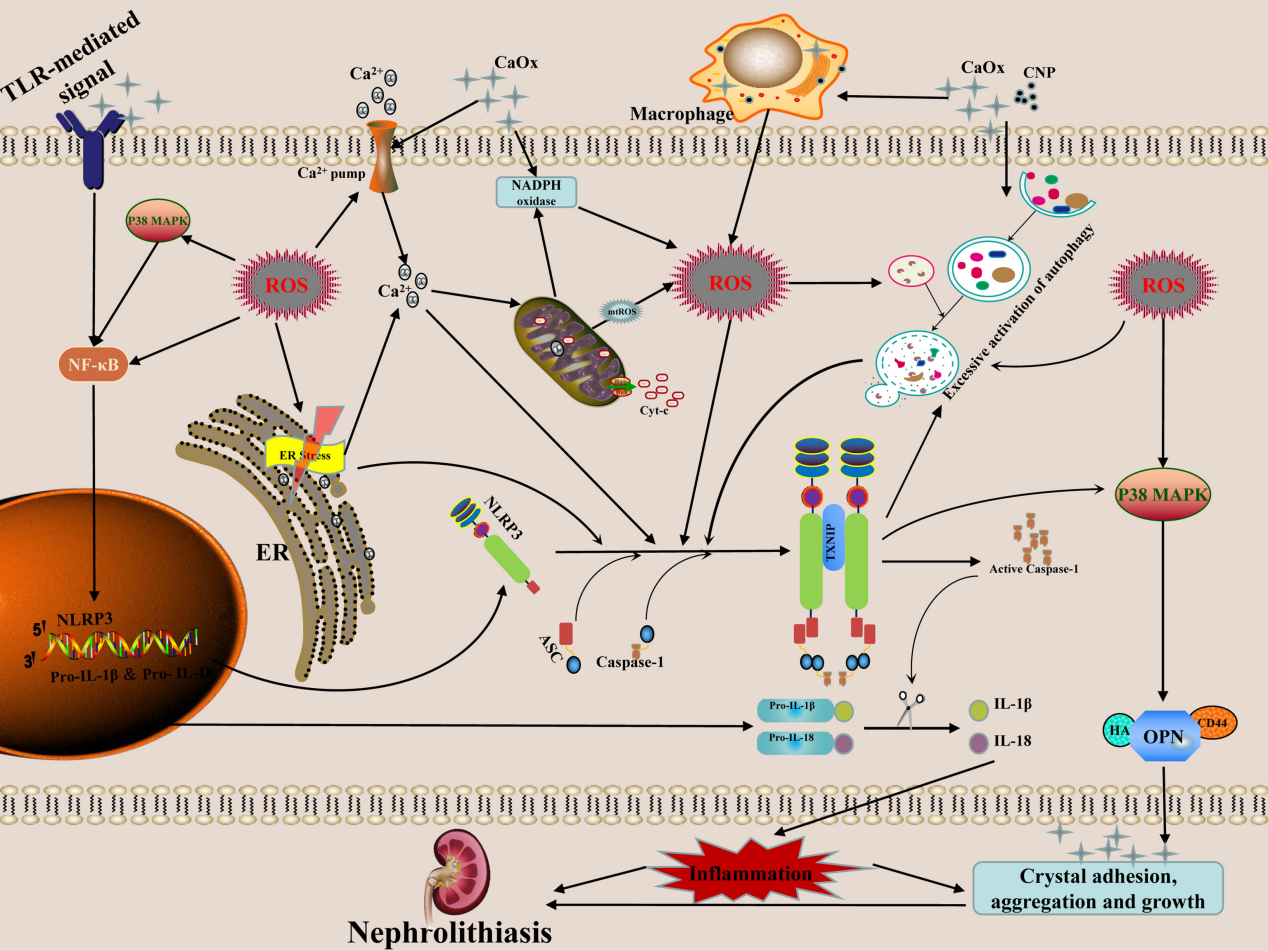
Possible mechanism and regulation of ROS-induced NLRP3 inflammasome activation in the formation of calcium oxalate nephrolithiasis. The secretion of mature forms of IL-1β and IL-18 is the result of NLRP3 inflammasome activation. These mediators have the properties of pro-inflammatory activation, which in turn promote the adhesion, aggregation and growth of crystals. The interaction of crystals or nanoparticles with cells can cause mitochondrial damage and increased NADPH oxidase activity, and then generate ROS, which mediates NLRP3 inflammasome transcription and activation through the ROS-dependent of NF-κB and autophagy signaling pathway. ER induces ROS production *via* NOX4 and ERO1 during stress. The release of Ca2+in the ER causes mitochondrial damage which further aggravates the release of ROS. High concentration of ROS can induce excessive activation of autophagy, thereby stimulate the inflammasomes to release a great number of inflammatory factors. The activation of NLRP3 inflammasome may further increase the level of autophagy, resulting in an inflammatory chain reaction.

In recent years, with the development of modern biology technology, the role of the cell-crystal inflammatory response theory in the formation of CaOx kidney stones has been increasingly valued. A growing body of literature suggests that the RTECs-crystal reaction, macrophage-crystal reaction, calcifying nanoparticles, ER stress, autophagy activation, and other regulatory factors can induce ROS production during the course of CaOx kidney stones formation. These processes can also mediate the activation of the NLRP3 inflammasome, promote the release of inflammatory factors such as IL-1β andIL-18, cause inflammatory cell infiltration and renal degeneration of tubular epithelial cells, necrosis, the kidney inflammatory cascade effect, hence stimulating the adhesion, aggregation, nucleation, and subsequent growth of CaOx crystals, and ultimately the formation of CaOx nephrolithiasis (**Figure 1**). In conclusion, effective intervention for ROS-induced activation of NLRP3 inflammasome may be a potential therapeutic target in the prevention of CaOx kidney stones formation and recurrence, which has important theoretical significance and practical value. 

## Data Availability Statement

The original contributions presented in the study are included in the article/supplementary material. Further inquiries can be directed to the corresponding author.

## Author Contributions

HX, YD, and JK contributed to conception and design of the study. YL wrote the first draft of the manuscript. YS, ZH, QL, JW, DL, XW, ZT, XG, and WS wrote sections of the manuscript. All authors contributed to manuscript revision, read, and approved the submitted version.

## Funding

This work was supported by National Natural Science Foundations of China (81960138, 81760127, 81360113, 30960455 and 30860280) and the Scientific Research and Technology Development Program of Guangxi (AB16380225).

## Conflict of Interest

The authors declare that the research was conducted in the absence of any commercial or financial relationships that could be construed as a potential conflict of interest.

## Publisher’s Note

All claims expressed in this article are solely those of the authors and do not necessarily represent those of their affiliated organizations, or those of the publisher, the editors and the reviewers. Any product that may be evaluated in this article, or claim that may be made by its manufacturer, is not guaranteed or endorsed by the publisher.

## Abbreviations

RTECs, The renal tubular epithelial cells; Ox, oxalate; CaOx, calcium oxalate; ROS, reactive oxygen species; NADPH, nicotinamide adenine dinucleotide phosphate; MDA, malondialdehyde; TBARS, thiobarbituric acid reactive substances; α-GST, α-glutathione-s-transferase; GAL, β-galactosidase; NAG, N-acetyl-β-D-glucosaminidase; NAC, N-acetylcysteine; SOD, superoxide dismutase; DAMPs, dangerous-associated molecular patterns; PAMPs, pathogen-associated molecular patterns; NLRs, NOD-like receptors;ASC, apoptosis-associated spot-like protein; HA, hyaluronic acid; OPN, osteopontin; TNF-α, tumor necrosis factor-α; CNPs, nanoparticles; HMGB1, High mobility group box-1; ER, endoplasmic reticulum; UPR, unfolded protein response; NOX, NADPH oxidase; ERO1, ER oxidase 1; MAPK, mitogen-activated protein kinase; PKR, protein kinase R; CHOP, C/EPB homologous protein; As_2_O_3,_ arsenic trioxide.
